# Modulation of Human Airway Barrier Functions during *Burkholderia thailandensis* and *Francisella tularensis* Infection Running Title: Airway Barrier Functions during Bacterial Infections

**DOI:** 10.3390/pathogens5030053

**Published:** 2016-08-03

**Authors:** Cornelia Blume, Jonathan David, Rachel E. Bell, Jay R. Laver, Robert C. Read, Graeme C. Clark, Donna E. Davies, Emily J. Swindle

**Affiliations:** 1 Academic Unit of Clinical and Experimental Sciences, Faculty of Medicine, University of Southampton, University Hospital Southampton, Tremona Road, Southampton SO16 6YD, UK; J.R.Laver@soton.ac.uk (J.R.L.); R.C.Read@soton.ac.uk (R.C.R.); D.E.Davies@soton.ac.uk (D.E.D.); E.J.Swindle@soton.ac.uk (E.J.S.); 2 Defence Science and Technology Laboratory (Dstl), Porton Down, Salisbury SP4 0JQ, UK; JDAVID@mail.dstl.gov.uk (J.D.); rachel.e.bell@kcl.ac.uk (R.E.B.); GCCLARK@dstl.gov.uk (G.C.C.); 3 Centre for Molecular and Cellular Biology of Inflammation, Guy’s Campus, King’s College London, London SE1 1UL, UK; 4 Southampton NIHR Respiratory Biomedical Research Unit, University Hospital Southampton, Southampton SO16 6YD, UK; 5 Institute for Life Sciences, University of Southampton, Southampton SO17 1BJ, UK

**Keywords:** airway epithelium, bacterial infection, *Burkholderia thailandensis*, *Fransicella tularensis*, barrier functions, bacterial passage, fluticasone propionate

## Abstract

The bronchial epithelium provides protection against pathogens from the inhaled environment through the formation of a highly-regulated barrier. In order to understand the pulmonary diseases melioidosis and tularemia caused by *Burkholderia thailandensis* and *Fransicella tularensis*, respectively, the barrier function of the human bronchial epithelium were analysed. Polarised 16HBE14o- or differentiated primary human bronchial epithelial cells (BECs) were exposed to increasing multiplicities of infection (MOI) of *B. thailandensis* or *F. tularensis* Live Vaccine Strain and barrier responses monitored over 24–72 h. Challenge of polarized BECs with either bacterial species caused an MOI- and time-dependent increase in ionic permeability, disruption of tight junctions, and bacterial passage from the apical to the basolateral compartment. *B. thailandensis* was found to be more invasive than *F. tularensis*. Both bacterial species induced an MOI-dependent increase in TNF-α release. An increase in ionic permeability and TNF-α release was induced by *B. thailandensis* in differentiated BECs. Pretreatment of polarised BECs with the corticosteroid fluticasone propionate reduced bacterial-dependent increases in ionic permeability, bacterial passage, and TNF-α release. TNF blocking antibody Enbrel^®^ reduced bacterial passage only. BEC barrier properties are disrupted during respiratory bacterial infections and targeting with corticosteroids or anti-TNF compounds may represent a therapeutic option.

## 1. Introduction

The lung epithelium forms the first line of defence against inhaled pathogens and, through regulation of epithelial barrier functions, it is a key player in maintaining tissue homeostasis [1]. Key components in anti-microbial defence are secreted by bronchial epithelial cells (BECs) into the mucus layer and include defensins, nitric oxide (NO), and reactive oxygen species (ROS) [2]. The mucus layer itself is mainly composed of hydrated mucins that provide viscosity and enables entrapment of bacteria, which are transported out of the lungs to the hypopharynx via the action of ciliated epithelial cells within the mucociliary escalator. In the healthy lung, commensal bacteria are adapted to these epithelial defence mechanisms and a stable microflora can develop without causing any inflammation. However, some pathogenic bacteria can overcome the anti-microbial defence mechanisms of the epithelium and cause symptomatic infections [3].

*Francisella tularensis* is a Gram-negative, pleomorphic, aerobic coccobacillus and is the causative agent of the disease tularaemia. Tularaemia is endemic in Scandinavia and North America with 30%–60% mortality following exposure to the most pathogenic strains, if left untreated [4]. Symptoms of pneumonic tularaemia include coughing, chest pains, and difficulty breathing. Aerosolised *F. tularensis* is the most likely candidate for use as a bioweapon [5]. With as few as 10 colony-forming units able to cause serious disease [4] this bacterium is placed on the Center for Disease Control and Prevention (CDC) Category A list of agents that pose a risk to national security and, as such, is handled at Advisory Committee on Dangerous Pathogens (ACDP) Level 3 [6]. An attenuated subspecies known as *F. tularensis* subsp. *holarctica* Live Vaccine Strain (LVS) is often used to enable higher throughput studies at ACDP Level 2 and shares many characteristics with more virulent strains [7]. *Burkholderia pseudomallei* is endemic in Thailand and Australia and causes melioidosis with non-specific symptoms leading to septic shock and high mortality rates if left untreated [8,9]. Meliodosis results from an acute or latent infection with the longest recorded period of latency some 62 years [10]. Therapeutic options for melioidosis are limited with no licensed vaccine and antibiotics being required for many months. For *Burkholderia pseudomallei* the ACDP2 model organism *Burkholderia thailandensis* has a reduced virulence (~10^5^ fold) [11] but is genetically similar to *B. pseudomallei* sharing 95% 16S rRNA similarity and virulence factor homologues [12]. By using these less virulent strains and by understanding their limitations it is possible to conduct mechanistic studies without the restrictions of working to ACDP3. Both tularaemia and melioidosis are most severe after exposure by the inhalation route and as such their interaction with the lung epithelium remains an important area of study.

The majority of experimental studies investigating respiratory infections are performed in animals, mainly mice. Animal models have several shortfalls and the translation of findings into the human situation can be difficult. As an alternative to animal models in respiratory research, human cell-based ex vivo and in vitro models have been developed [13]. However, there are only a limited number of studies published that use human in vitro models to investigate epithelial-bacterial interactions. A variety of human lung epithelial cell lines are used in respiratory research, but only a few are able to express tight junction complexes under certain culture conditions which are an essential part of the physical barrier of the epithelial sheet. Tight junctions also maintain the polarity of the epithelium and distinguish between the lumen-facing apical, and the ‘tissue-facing’ basolateral compartment. 

Despite their advantages, cell line models of the polarised airway epithelium have some limitations as they do not express all components of the epithelial barrier functions observed in vivo, such as ciliated cells and mucus-producing goblet cells. An in vitro model that recapitulates the in vivo situation more closely utilises human primary bronchial epithelial cells (PBECs) which can be differentiated to form a pseudostratified mucociliary epithelium when grown at the air-liquid interface on semi-permeable membrane supports. While these in vitro cultures are expensive to maintain, and results can be variable due to the phenotypic differences of the donors, they are invaluable for determination of the response of the human airway epithelial barrier to infection. Furthermore, such in vitro models allow higher throughput preliminary evaluation of therapeutics, making them ideal for early drug discovery.

The aim of this study was to analyse the effect of *B. thailandensis* and *F. tularensis* LVS infection on airway epithelial barrier properties. We show that *B. thailandensis* and *F. tularensis* both disrupt the physical barrier resulting in their passage from the apical to the basolateral epithelial surface and activation of the immunological barrier with induction of TNF-α release. We confirm these findings utilising fully-differentiated primary epithelial cells exposed to *B. thailandensis.* Additionally, we demonstrate the use of this model to identify novel therapeutic candidates for the treatment of respiratory pathogens.

## 2. Results

### 2.1. Bacterial Infection Alters the Physical Barrier Properties of the Human Bronchial Epithelium

Prior to exposing epithelial cells to bacteria, growth of the bacteria in epithelial culture medium was assessed. Both *B. thailandensis* and *F. tularensis* were able to proliferate in airway epithelial culture medium (Figure S1), however, *F. tularensis* LVS showed slower growth rates in the first 5 h (up to two-fold OD increase) compared to *B. thailandensis* (5 to 6-fold OD increase). 

**Figure 1:**
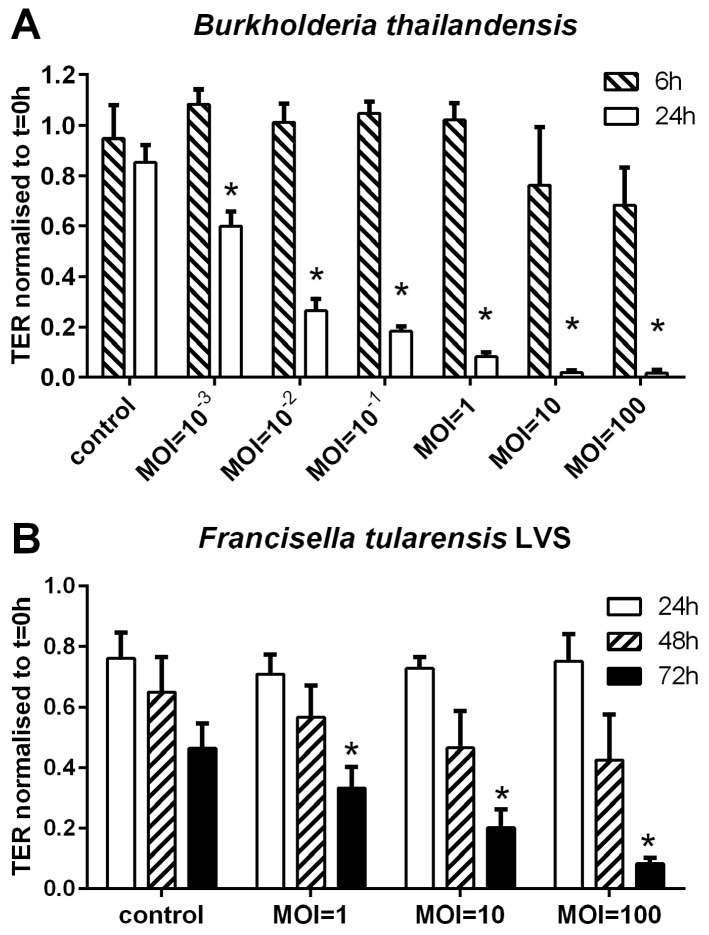
*B. thailandensis* and *F. tularensis* LVS disrupt the physical barrier properties of bronchial epithelial cells. Polarised 16HBE cells were infected apically with a bacterial suspension of indicated multiplicity of infection (MOI) and the ionic permeability determined by measuring transepithelial resistance (TER). (**A**) Infection of polarised 16HBE cells with *B. thailandensis* (MOI from 10^−3^ to 10^2^). TER was measured after 6 h and 24 h of infection; (**B**) Polarised 16HBEs were infected with *F. tularensis* (MOI from 1 to 100). TER was analysed 24 h, 48 h and 72 h after infection. Results are normalised to t = 0 h after subtraction of the background TER of an empty transwell. Means ± SEM, *n* = 3–14 (**A**) and *n* = 3 (**B**). * *p* ≤ 0.05 compared to control.

Following infection of polarised BECs with *B. thailandensis,* there was an increase in the ionic permeability as measured by a decrease in transepithelial resistance (TER). This effect was multiplicity of infection (MOI)- and time-dependent (Figure 1A). While no significant change in the TER was observed within 6 h of infection, a decrease was observed (60% of pre-infection baseline control) after 24 h at a MOI of 10^−3^. At MOIs of greater than, or equal to, 1 there was an almost complete loss of electrical barrier integrity. This response may be caused by an increase in cell death. In contrast, infection of polarised 16HBE cells with *F. tularensis* LVS resulted in a much slower increase in the ionic permeability even at higher initial infection doses (Figure 1B). However, the TER was significantly reduced after 72 h of infection at a MOI of 1 and above. Reduction of TER to background levels was only achieved after 72 h of infection at the highest MOI tested (MOI = 100).

**Figure 2:**
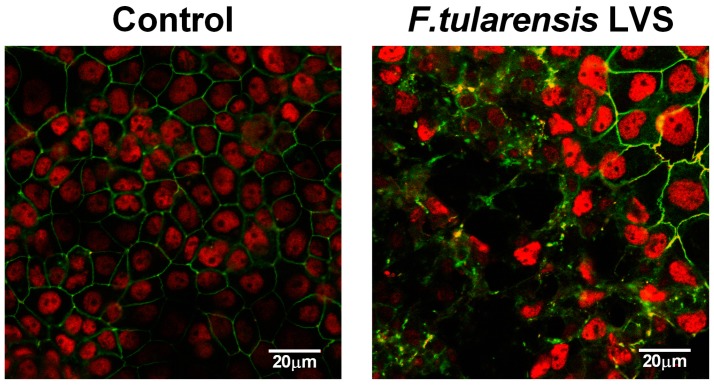
Disruption of tight junctions in bronchial epithelial cells after *F. tularensis* infection. Polarised 16HBE cells were apically infected with *F. tularensis* LVS (MOI of 1) for 72 h and distribution of the tight junction protein occludin (green) analysed by confocal fluorescence microscopy. Nuclei stained with DAPI were shown in pseudo-colouring (red) representative image of three independent experiments.

Since infection with *F. tularensis* showed a slower and graduated response than *B. thailandensis* we analysed the expression and localisation of the tight junction protein occludin in *F. tularensis* infected BECs by confocal fluorescence microscopy. As shown in Figure 2, the ring-like distribution of occludin at cell-cell contacts observed in uninfected polarised BECs was disrupted after infection with *F. tularensis.* It appears that integrity is lost in specific areas rather than across the whole epithelial layer. The disruption correlates with the TER measurements, where we observed only a partial drop after 72 h of infection at a MOI of 1.

### 2.2. Passage of Bacteria across the Airway Barrier

**Figure 3:**
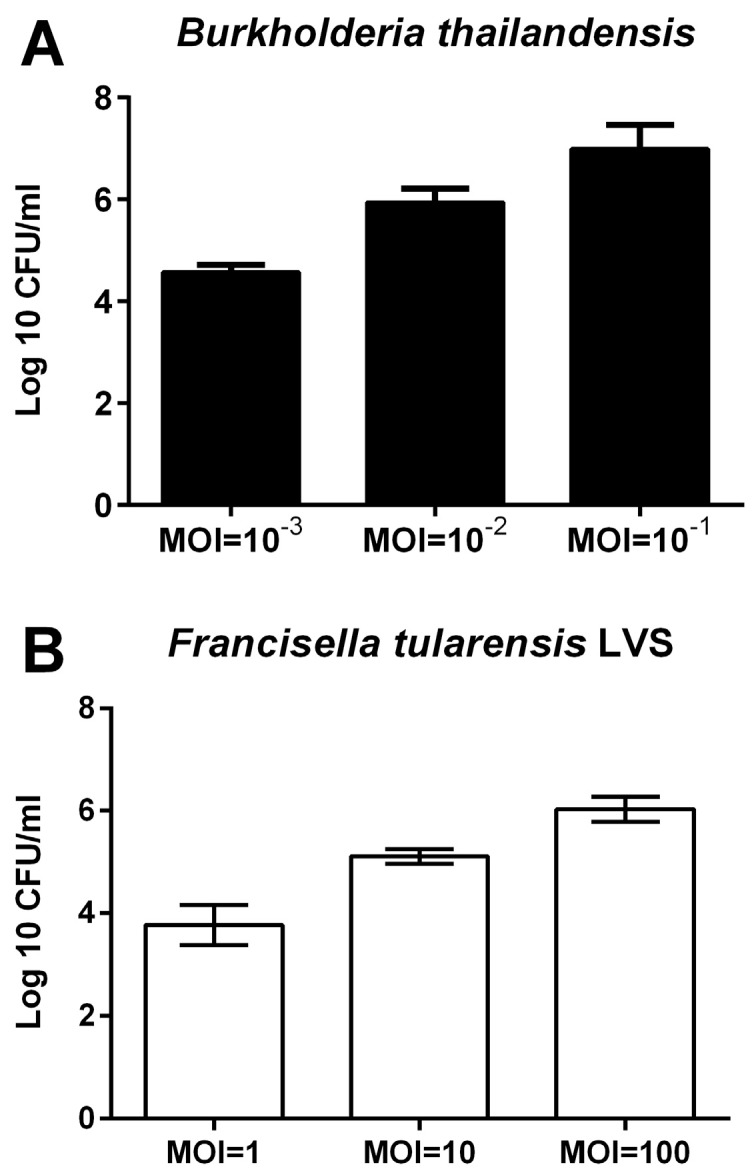
Passage of bacteria across the epithelial barrier. Polarised 16HBE cells were apically infected with bacteria and the number of bacteria crossing the epithelial barrier assessed by counting live bacteria in the basolateral compartment. (**A**) Passage of *B. thailandensis* across the epithelial barrier after 24 h of infection; and (**B**) epithelial passage of *F. tularensis* LVS after 72 h of infection. Results are means ± SEM, *n* = 3.

Since infection of polarised BECs with *B. thailandensis* or *F. tularensis* LVS resulted in an increased ionic permeability, we analysed whether the bacteria were able to penetrate to the basolateral compartment of the epithelial culture. Counting of live bacteria showed that both bacterial strains were able to cross the epithelial barrier (Figure 3); passage of bacteria across the epithelial barrier correlated with the initial infection dose and the gradual loss of the physical barrier properties and corresponding reduction in TER. 

### 2.3. B. thailandensis and F. tularensis LVS Activate Immunological Airway Epithelial Barrier Function

**Figure 4:**
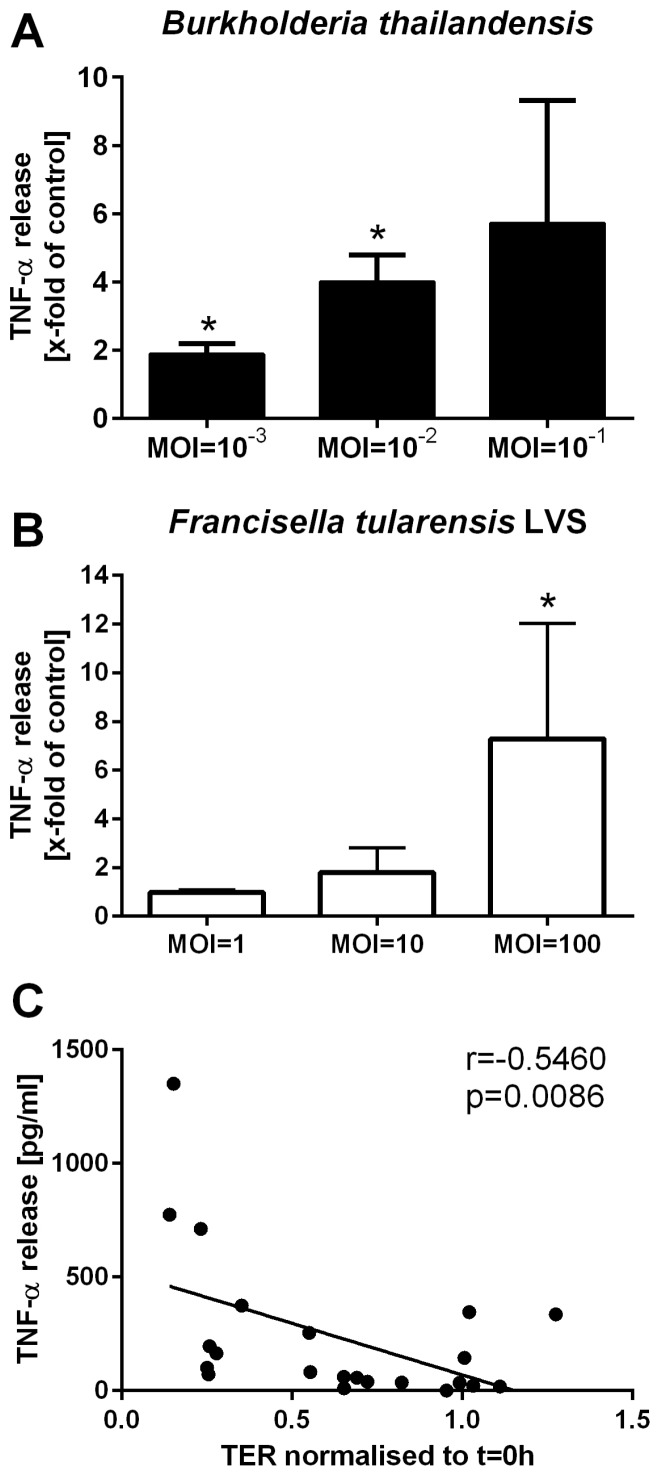
*B. thailandensis* and *F. tularensis* infection of airway epithelial cells activated the immunological barrier functions. Polarised 16HBE cells were apically infected with *B. thailandensis* or *F. tularensis* and the basolateral release of inflammatory mediators was analysed. Basolateral release of TNF-α after 24 h of infection with *B. thailandensis* by analysed by ELISA (**A**) and, after 72 h of *F. tularensis* LVS infection, by CBA assay (**B**) normalised to untreated control (A: untreated control: 85.3 ± 45.4 pg/mL; B: 2.4 ± 1.2 pg/mL). Results are means ± SEM, *n* = 3–6 (**A**) and *n* = 3 (**B**). * *p* ≤ 0.05 compared to control; and (**C**) correlation of TNF-α release with ionic permeability determined by measuring transepithelial resistance (TER).

Infection with *B. thailandensis* or *F. tularensis* LVS was also found to trigger an inflammatory response by BECs. Polarised 16HBE cells released TNF-α into the basolateral compartment after infection with *B. thailandensis* or *F. tularensis* LVS in a MOI-dependent manner (Figure 4A,B). Increased release of TNF-α was associated with the observed increase in ionic permeability of the epithelial cell layer (Figure 4C).

### 2.4. B. thailandensis Alters Barrier Functions of Differentiated Human Primary Bronchial Epithelial Cells (PBECs)

**Figure 5:**
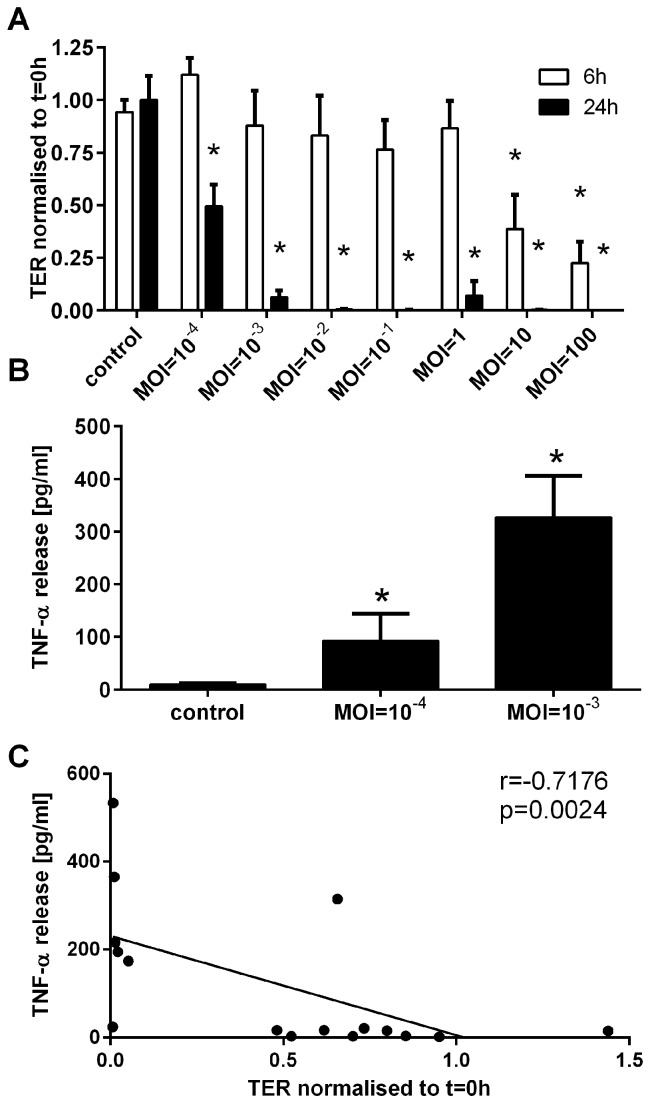
Differentiated PBECs are more sensitive to infection with *B. thailandensis*. After apical infection of differentiated PBECs with *B. thailandensis*, physical and immunological barrier properties were monitored. (**A**) Ionic barrier permeability was measured by TER after 6 h and 24 h of infection and normalised to t = 0 h after subtraction of the background TER of an empty TW; (**B**) basolateral release of TNF-α after 24 h of infection was analysed by ELISA; and (**C**) correlation of TNF-α release with ionic permeability determined by measuring the transepithelial resistance (TER). Results are means ± SEM, *n* = 3–6 (**A**) and *n* = 4–6 (**B**). * *p* ≤ 0.05 compared to control.

To validate the data generated in the polarised 16HBE cell model, differentiated human primary bronchial epithelial cells (PBECs) were apically infected with *B. thailandensis.* Using differentiated PBECs, the ionic permeability of the epithelial barrier was significantly increased after 6 h of infection at the highest MOIs of 10 and 100 of *B. thailandensis*, whilst after 24 h of infection the ionic permeability was significantly increased at all MOIs tested (Figure 5A). After 24 h of infection the TER was reduced by around 50% at a MOI of 10^−4^ whereas, at higher MOIs, there was a complete loss in electrical barrier integrity. Similar to the cell line model, infection of differentiated PBECs caused a significant increase in basolateral release of TNF-α (Figure 5B), which correlated with the breakdown of the physical barrier properties (Figure 5C). Since fully-differentiated PBECs express many antimicrobial defence mechanisms, including beating cilia and mucus production that are absent in the polarised cell line model, we postulated that differentiated PBECs are more resistant to *B. thailandensis* infection. Surprisingly, differentiated PBECs were more sensitive to *B. thailandensis* infection with an MOI of 10^−3^ causing a complete loss of electrical barrier integrity and a significant increase in TNF-α release, whereas the 16HBE cell line model was only modestly affected at this MOI.

### 2.5. Pharmacological Manipulation of Epithelial Barrier Functions

**Figure 6:**
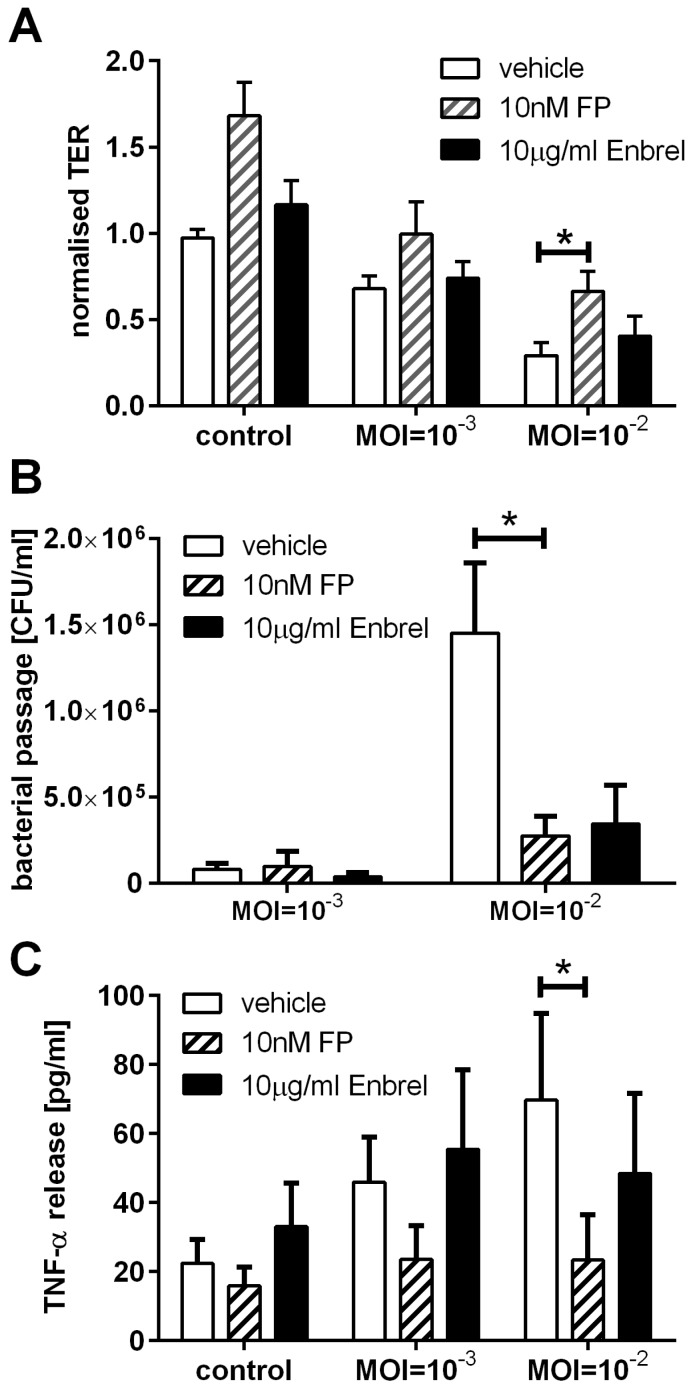
Corticosteroids and anti-TNF-α treatment alter epithelial barrier functions during *B. thailandensis* infection. Polarised 16HBEs were pre-treated with 10 nM fluticasone propionate (FP) or 10 μg/mL anti-TNF-α (Enbrel^®^) for 1 h before apical infection with *B. thailandensis* for 24 h. (**A**) Physical barrier properties measured by TER are normalised to t = 0 h; (**B**) passage of bacteria across the epithelial barrier are determined by bacterial counts in the basolateral medium; and (**C**) basolateral release of TNF-α measured by ELISA. Results are means ± SEM, *n* = 5. * *p* ≤ 0.05.

The addition of corticosteroids or anti-TNF-α antibodies have previously been shown to influence physical and immunological barrier functions of the airway epithelium [14,15,16]. We, therefore, analysed the effect of the corticosteroid fluticasone propionate, which is used as an inhaled corticosteroid to treat excessive airway inflammation in asthma, and the TNF-α neutralising drug Enbrel^®^ (etanercept, soluble TNFα receptor Fc fusion protein) on *B. thailandensis*-induced activation of airway barrier functions in the 16HBE cell model. While the TNF-α binding drug Enbrel only partially reduced the drop in TER, fluticasone propionate significantly counteracted the reduction in TER after 24 h of infection with *B. thailandensis* at the MOI of 10^−2^ (Figure 6A). The passage of bacteria across the epithelial barrier was also reduced by fluticasone propionate or by neutralisation of TNF-α, however, the effect was only statistically significant for fluticasone propionate (Figure 6B). In addition, treatment with fluticasone propionate caused a significant reduction in *B. thailandensis* induced TNF-α release (Figure 6C).

## 3. Discussion

In this study we demonstrate that the polarised cell line 16HBE14o- is a useful in vitro model of the airway epithelium for studying the effect of *B. thailandensis* and the slower growing *F. tularensis* on epithelial barrier functions. Both bacteria are able to diminish the physical barrier properties, although in these models the airway epithelium is more susceptible to *B. thailandensis* than to *F. tularensis* LVS. The passage of bacteria across the epithelial barrier is linked to the breakdown of the physical barrier. Both bacterial strains induce a proinflammatory response with the induction of TNF-α release in a MOI-dependent manner. Furthermore, the activation of the airway epithelial barrier functions induced by *B. thailandensis* infection are reduced by the corticosteroid fluticasone propionate resulting in improved physical barrier properties, reduced bacterial passage, and decreased release of TNF-α.

The interaction of different genera of bacteria with the airway epithelium has been analysed previously using in vitro cell culture or animal models [17]. In the lung, epithelial cells are the first line of defence and express a variety of pathogen recognition receptors (PRRs) including toll-like receptors (TLRs) [18,19]. Important PRRs for bacterial cell wall components, like lipopeptides or lipopolysaccharides (LPS), are TLR2 and 4. Binding of bacterial products to TLR2 or 4 results in downstream activation of NF-κB and p38 mitogen-activated protein kinase (MAPK) signalling pathways [18,20], which are involved in the regulation of the physical but also the immunological barrier functions of airway epithelial cells. Interestingly, it has been shown that *Burkholderia cenocepacia* activates an inflammatory response in airway epithelial cells via TLR5 independent of TLR2/4 signalling [21]. Additionally, a breakdown of the physical barrier can be mediated independently of PPR signalling by the release of bacterial virulence factors; for example, type III toxins of *Pseudomonas aeruginosa* interfering with cytoskeletal organisation [22] or hemolysin A, a pore-forming protein, released by *Staphylococcus aureus* [23]. 

Frequently, a breakdown of the epithelial physical barrier is correlated with an increase in paracellular permeability of macromolecules, as well as particles, including the transmigration of bacteria into the interstitium. An increased passage of bacteria across the airway epithelial sheet has been shown previously using in vitro models with various bacterial strains. For example, infection of polarised 16HBE cells with *Staphylococcus aureus* increased the passage across the epithelial layer, a process that is thought to be mediated by the bacterial surface protein A [24]. The type III toxins of *Pseudomonas aeruginosa* are also associated with increase bacterial passage across the epithelium [22]. Other bacterial virulence factors like rhamnolipids released by *P. aeruginosa* interfere with the physical barrier properties of the airway epithelium and enhance the invasion of bacteria across the epithelial barrier [25]. Furthermore, bacterial activation of TLR2, p38 MAPK, and TGF-β signalling pathways are involved in the bacteria-induced barrier disruption and increased passage of bacteria across the epithelial layer [20]. In contrast, studies using gut epithelial models, suggest that commensal and probiotic bacteria have protective effects on epithelial barrier function [26] indicating that comparative work on airway commensals would be of interest. 

An important role of the airway epithelium in response to bacterial infections is the induction of inflammation, which is mediated by the release of inflammatory mediators. Airway epithelial cells are able to release a variety of pro- and anti-inflammatory mediators [1] orchestrating the infiltration and activation of immune cells in the airway mucosa. Important immune cells required for bacterial clearance are macrophages and neutrophils, which are attracted and activated to sites of infection by IL-8, IL-6, and TNF-α [27]. Here we show that *B. thailandensis* and *F. tularensis* both induce the release of TNF-α by airway epithelial cells, which can activate an inflammatory response. 

In the airway mucosa at sites of bacterial infection, infiltrating activated immune cells release a variety of anti-microbial molecules, including ROS and NO. However, excessive inflammation can result in tissue damage and facilitate systemic infection. Therefore, regulating excessive airway inflammation and a breakdown of the physical barrier properties during bacterial infections is one of the most promising therapeutic strategies despite the use of antibiotics. In this study we investigated the effect of corticosteroids and anti-TNF-α therapy on the physical and immunological barrier properties of airway epithelial cells. TNF-α has been shown to mediate a disruption of the physical barrier of airway epithelial cells [14], which might increase the passage of bacteria across the epithelial barrier and contribute to systemic infection. The breakdown of the physical barrier properties of the airway epithelium during bacterial infection was reduced by administrating a TNF-α binding drug leading to a decrease in bacterial passage across the epithelium. Administration of anti-TNF-α drugs during bacterial airway infections might help reduce the risk of systemic infections.

The corticosteroid fluticasone propionate improved the physical barrier properties, decreased the passage of bacteria across the epithelium, and reduced the release of inflammatory mediator by the airway epithelium during bacterial infection. Corticosteroids have previously been shown to improve the physical barrier properties of airway epithelial cells [15]; however, their effect on bacterial passage across the airway epithelium during bacterial infections has not been analysed. Additionally, the effects of corticosteroids on airway epithelial functions involved in innate and adaptive immunity are well known [28]. Our data showing reduced release of TNF-α during *B. thailandensis* infection by corticosteroid treatments are in line with previously published data for other respiratory pathogens. In these prior studies using an airway epithelial cell line in monolayer culture, it has been shown that fluticasone propionate inhibits *S. aureus*-induced IL-8, IL-6, and TNF-α expression in airway epithelial cells by reducing AP-1 and NF-κB signalling [29]. 

Reduction of the inflammatory response of airway epithelial cells during bacterial infections has the potential to prevent excessive inflammation in the airways that can ultimately cause severe tissue damage, septicaemia, and lung failure. Here we demonstrate the use of human epithelial cell models in order to evaluate putative therapeutics that aid in the maintenance of lung barrier function during the course of an infection. The administration of corticosteroids or anti-TNF-α drugs represent potential therapeutic strategies that maintain the physical barrier functions of the lung and reduce the risk of excessive inflammation during lung infections with pathogenic bacteria, such as *B. thailandensis*.

## 4. Experimental Section

### 4.1. Cell Culture

The human bronchial epithelial cell line 16HBE14o- (16HBE; a gift from Prof. D.C. Gruenert, San Francisco, CA, USA) and differentiated primary human bronchial epithelial cells (PBECs) were used in this study. 16HBE cells were maintained in minimum essential medium (MEM) with Glutamax and supplemented with 10% foetal bovine serum and penicillin/streptomycin (Life technologies, Paisley, UK) on PureCol collagen I (Advanced BioMatrix, San Diego, CA, USA) coated culture flasks. Experiments were carried out using collagen-coated Transwell^®^ permeable supports (diameter 6.5 mm, polyester membrane with 3 µm pores, Corning Life Sciences, Amsterdam, The Netherlands). Cells were seeded at a density of 1.5 × 10^5^ cells in 200 µL growth medium; the basolateral compartment contained 500 µL of the same medium. Medium exchange was carried out every 2–3 days. 16HBE cells formed a polarised epithelial sheet within seven days of culture, as monitored by measuring the transepithelial resistance (TER) using an EVOM voltohmmeter (World Precision Instruments, Hitchin, UK). Cells with a TER <330 Ω·cm^2^ on day 7 were used for experiments.

Differentiation of human PBECs at the air-liquid interface (ALI) was performed as previously described [30]. Briefly, PBECs were obtained by epithelial brushings using fiber optic bronchoscopy from healthy subjects selected from a volunteer database. All procedures were approved by the Southampton and South West Hampshire Research Ethics Committee and were undertaken following informed consent. PBECs were cultured in bronchial epithelial growth medium (BEGM; Lonza, Basel, Switzerland). At passage 2, cells were cultured on collagen coated Transwell^®^ permeable supports (diameter 6.5 mm, polyester membrane with 0.4 µm pores, Corning Life Sciences, Amsterdam, The Netherlands) and differentiation was induced by ALI culture for 21 days. TER was monitored weekly and cells with a TER <330 Ω·cm^2^ on day 21 were used for experiments.

### 4.2. Bacterial Culture

*B. thailandensis* strain E264 [31] was grown in liquid LB medium at 37 °C, 5% CO_2_ under agitation. Bacterial growth was assessed by measuring the optical density at OD_600nm_. For determination of live bacteria counts, serial dilutions of the bacterial suspension culture were plated on LB agar plates, incubated for 24 h and the colony-forming unit (CFU) per mL liquid medium calculated.

The live attenuated strain of *F. tularensis* (LVS) was reconstituted from *Pasteurella tularensis* Vaccine Batch Lot 4 (National Drug Co., Swiftwater, PA, USA). All stocks and working cultures were handled in accordance with ACDP containment level 2 requirements. Bacteria were resuscitated from frozen stock cultures by overnight growth at 37 °C on BCGA plates. For infection assays spectrophotometric measurements were taken of bacteria resuspended in PBS where an absorbance of 0.2 at 590 nm equated to 1 × 10^9^ cfu/mL. Enumeration of bacteria from all infection experiments were carried out using a logarithmic serial dilution of samples in PBS cultured on BCGA plates in triplicate at 37 °C for 48 h.

### 4.3. Infection of Airway Epithelial Cells

Cells were cultured in antibiotic free medium 24 h before and throughout the experiments. After measuring the TER (t = 0 h), the apical medium of airway epithelial cells was removed and replaced with 100 µL of bacterial suspension at indicated multiplicity of infection (MOI). Cells were incubated for 60 min at 37 °C and, subsequently, the apical bacterial solution was removed. In experiments performed with polarised 16HBE cells, 200 µL of fresh medium was added to the apical compartment while differentiated PBECs were cultured at the air-liquid interface for the duration of the experiment. The TER was measured after indicated time points. The background TER of an empty transwell (170 Ω) was subtracted and TER data were normalised to the measurements before infection at t = 0 h. After taking apical and basolateral supernatants, bacterial live counts were determined, and the supernatants stored at −20 °C until further analysis.

The effect of the glucocorticoid fluticasone propionate (Sigma Aldrich, Gillingham, UK) and the TNF-α binding drug Enbrel (Wyeth Europa Ltd., Maidenhead, UK) was analysed on airway epithelial barrier functions during bacterial infection. Polarised 16HBE cells were pre-incubated with 10 nM FP or 10 μg/mL Enbrel in the apical and basolateral medium for 60 min prior infection. Apical supernatants were removed and replaced by the *B. thailandensis* suspension at indicated MOIs without inhibitors. After incubation for 60 min at 37 °C the apical bacterial solution was removed and 200 µL of fresh medium containing inhibitors was added to the apical compartment. 

### 4.4. Confocal Laser Scanning Microscopy

Visualisation of cellular junctions was performed using confocal microscopy. Cells were seeded onto 10 mm diameter glass coverslips (VWR International Ltd., Lutterworth, UK) in 24-well plates. At experimental time-points cells were fixed with 1 mL of 4% *w*/*v* paraformaldehyde (Pioneer Research Chemicals, Colchester, UK) before being permeabilised with 0.2% saponin). Cells were quenched with 50 mM ammonium chloride in PBS for ten minutes before being blocked in 0.2% *w*/*v* gelatine), 0.02% saponin, 0.02% *w*/*v* sodium azide (all Sigma) in PBS (defined as PGAS) overnight at 4 °C. Coverslips were then incubated with 50 μL (10 μg/mL) IgG polyclonal antibodies to the tight junction protein occludin (Life Technologies, Paisley, UK) for 1 h at room temperature, washed three times with 1 mL PGAS and incubated with 50 μL (5 μg/mL) AlexaFluor^®^488 goat-anti-rabbit polyclonal antibodies (Life Technologies) for a further one hour in the dark at room temperature. After washing three times with 1 mL PGAS coverslips were mounted in UltraCruz mounting media containing DAPI (Santa Cruz Biotechnology Inc., Dallas, TX, USA). Fluorescence was assessed using an Olympus IX70 confocal microscope (Olympus, Hamburg, Germany) fitted with an Olympus Fluoview filter unit and Omnichrome ion laser power pack. All images were observed at 600× magnification and image capture was carried out using FluoView software (Olympus). Occludin stain is shown in green, whereas nuclei are shown in red.

### 4.5. Release of Inflammatory Mediators

In experiments with *B. thailandensis*-infected airway epithelial cells the basolateral release of TNF-α was determined by ELISA using a human TNF-α DuoSet ELISA kit (R&D Systems, Abingdon, UK). The release of basolateral TNF-α of airway epithelial cells infected with *F. tularensis* LVS was analysed using a Cytometric Bead Array (BD Biosciences, Oxford, UK) following the manufacturer’s instructions. 

### 4.6. Statistical Analysis

Statistical evaluation was performed using the software SigmaPlot 12.5. After testing for normality using the Kolmogorov-Smirnov test, related samples were analysed for statistical significance using the paired Student’s *t*-test or the non-parametric Wilcoxon test. Differences were regarded as significant when *p* ≤ 0.05.

## 5. Conclusions 

Using an in vitro model of the human airway epithelium we demonstrated that infection with *B. thailandensis* or *F. tularensis* activated airway epithelial barrier functions. Both bacteria caused a time- and multiplicity of infection-dependent breakdown of the physical barrier properties, which correlated with an increase in bacterial passage across the epithelial layer. Both bacteria also activated the immunological barrier with the release of TNF-α. The administration of corticosteroids or an anti-TNF-α drug improved the barrier properties of the airway epithelium during *B. thailandensis* infection with reduced bacterial passage and decreased inflammatory response. Targeting the airway epithelial barrier during *B.thailandensis* or *F. tularensis* infections with corticosteroids or anti-TNF compounds may represent potential therapeutic strategies for the treatment of the pulmonary diseases caused by bacterial infections, especially melioidosis and tularemia, by limiting bacterial passage across the epithelium and inflammation.

## Supplementary Materials

The following are available online at http://www.mdpi.com/2076-0817/5/3/53/s1. **Figure S1.** Bacterial growth in airway epithelial culture medium. *B. thailandensis* (**A**,**B**) and *F. tularensis* LVS (**C**,**D**) were grown in 16HBE medium or ALI medium with a starting optical density (OD) between 0.03 and 0.05. The OD (**A**,**C**) was measured over the first 5 h of culture and after 24 h the live bacteria counts were determined (**B**,**D**) as colony-forming units per mL culture medium (CFU/mL). Results are mean ± SD. *B. thailandensis*: *n* = 2; *F. tularensis* LVS: *n* = 3.
