# Erratum: Blume, C., et al. Modulation of Human Airway Barrier Functions during *Burkholderia thailandensis* and *Francisella tularensis* Infection Running Title: Airway Barrier Functions during Bacterial Infections. *Pathogens* 2016, *5*, 53

**DOI:** 10.3390/pathogens9120987

**Published:** 2020-11-26

**Authors:** Cornelia Blume, Jonathan David, Rachel E. Bell, Jay R. Laver, Robert C. Read, Graeme C. Clark, Donna E. Davies, Emily J. Swindle

**Affiliations:** 1 Academic Unit of Clinical and Experimental Sciences, Faculty of Medicine, University of Southampton, University Hospital Southampton, Tremona Road, Southampton SO16 6YD, UK; J.R.Laver@soton.ac.uk (J.R.L.); R.C.Read@soton.ac.uk (R.C.R.); D.E.Davies@soton.ac.uk (D.E.D.); E.J.Swindle@soton.ac.uk (E.J.S.); 2 Defence Science and Technology Laboratory (Dstl), Porton Down, Salisbury SP4 0JQ, UK; JDAVID@mail.dstl.gov.uk (J.D.); rachel.e.bell@kcl.ac.uk (R.E.B.); GCCLARK@dstl.gov.uk (G.C.C.); 3 Centre for Molecular and Cellular Biology of Inflammation, Guy’s Campus, King’s College London, London SE1 1UL, UK; 4 Southampton NIHR Respiratory Biomedical Research Unit, University Hospital Southampton, Southampton SO16 6YD, UK; 5 Institute for Life Sciences, University of Southampton, Southampton SO17 1BJ, UK

There is an error in the title. The correct title of the article [1] is “Modulation of Human Airway Barrier Functions during *Burkholderia thailandensis* and *Francisella tularensis* Infection”. We apologize for this error and state that the scientific conclusions are unaffected. The original article has been updated.
