# Supplementary material for: Modulation of Human Airway Barrier Functions during Burkholderia thailandensis and Francisella tularensis Infection Running Title: Airway Barrier Functions during Bacterial Infections
Source: Pathogens. 2016 Aug 3;5(3):53. doi: 10.3390/pathogens5030053 (PMC5039433; doi:10.3390/pathogens5030053)
Supplement: Supplementary file 1 [file pathogens-05-00053-s001.pdf]

Supplementary Materials: Modulation of Human Airway Barrier Functions during *Burkholderia thailandensis* and *Francisella tularensis* Infection Running Title: Airway Barrier Functions during Bacterial Infections

Cornelia Blume, Jonathan David, Rachel E. Bell, Jay R. Laver, Robert C. Read, Graeme C. Clark, Donna E. Davies and Emily J. Swindle

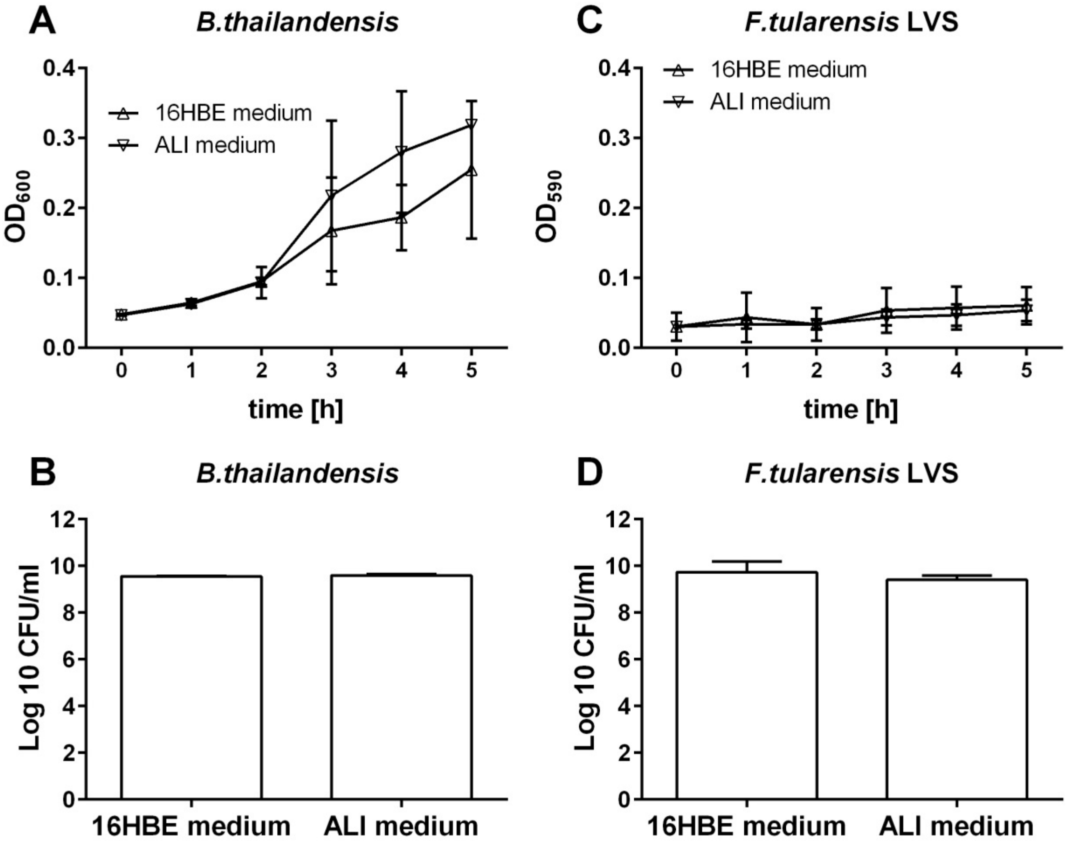

Figure S1. Bacterial growth in airway epithelial culture medium. *B. thailandensis* (**A**,**B**) and *F. tularensis* LVS (**C**,**D**) were grown in 16HBE medium or ALI medium with a starting optical density (OD) between 0.03 and 0.05. The OD (**A**,**C**) was measured over the first 5 h of culture and after 24 h the live bacteria counts were determined (**B**,**D**) as colony-forming units per mL culture medium (CFU/mL). Results are mean ± SD. *B. thailandensis*: $n = 2$; *F. tularensis* LVS: $n = 3$.
